# Natural compound bavachalcone promotes the differentiation of endothelial progenitor cells and neovascularization through the RORα-erythropoietin-AMPK axis

**DOI:** 10.18632/oncotarget.21036

**Published:** 2017-09-16

**Authors:** Shuang Ling, Rong-Zhen Ni, Yunyun Yuan, Yan-Qi Dang, Qian-Mei Zhou, Shuang Liang, Fujiang Guo, Wei Feng, Yuanyuan Chen, Katsumi Ikeda, Yukio Yamori, Jin-Wen Xu

**Affiliations:** ^1^ Institute of Interdisciplinary Medical Science, Shanghai University of Traditional Chinese Medicine, Shanghai, China; ^2^ Engineering Research Center of Modern Preparation Technology of Traditional Chinese Medicine, Shanghai University of Traditional Chinese Medicine, Shanghai, China; ^3^ School of Pharmacy, Shanghai University of Traditional Chinese Medicine, Shanghai, China; ^4^ School of Rehabilitation Medicine, Shanghai University of Traditional Chinese Medicine, Shanghai, China; ^5^ School of Pharmacy and Pharmaceutical Sciences, Mukogawa Women's University, Nishinomiya, Japan; ^6^ Institute for World Health Development, Mukogawa Women's University, Nishinomiya, Japan

**Keywords:** endothelial progenitor cell, bavachlcone, neovascularization, RORα, erythropoietin

## Abstract

In cardiovascular diseases, endothelial function is impaired and the level of circulating endothelial progenitor cells (EPCs) is low. This study investigated whether the natural bioactive component bavachalcone (BavaC) induces the differentiation of EPCs and neovascularization *in vivo*; the underlying mechanisms were also examined. We observed that the treatment of rat bone marrow–derived cells with a very low dose of BavaC significantly promoted EPC differentiation. In our hindlimb ischemia models, low–dose BavaC administered orally for 14 days stimulated the recovery of ischemic hindlimb blood flow, increased circulating EPCs, and promoted capillary angiogenesis. The BavaC treatment of rat bone marrow cells for 24 h initiated the AMP–activated protein kinase (AMPK) activity required for the differentiation of EPCs. Further testing revealed that BavaC and CGP52608, a retinoic acid receptor–related orphan receptor α (RORα) activator, enhanced the activity of RORα1 and EPO luciferase reporter gene. BavaC treatment also elevated EPO mRNA and protein expression *in vitro* and *in vivo* and the circulating EPO levels in rats. By contrast, the RORα antagonist VPR66 inhibited BavaC–induced EPO reporter activity, and differentiation of bone marrow cells into endothelial progenitor cells. Overall, this study revealed that BavaC promotes EPC differentiation and neovascularization through a RORα–EPO–AMPK axis. BavaC can be used as a promising angiogenesis agent for enhancing angiogenesis and tissue repair.

## INTRODUCTION

Erythropoietin (EPO), produced mainly in the renal interstitium, is a cytokine that stimulates erythrocyte differentiation [1–2]. EPO is also present in non-hematopoietic tissue, particularly in the brain and heart [3–5]. A previous study showed that EPO caused a significant mobilization of CD34^+^/CD45^+^ circulating endothelial progenitor cells (EPCs) in peripheral blood, and increased the number of functionally active EPCs [6]. EPO therapy has been widely applied to treat myocardial infarction [7–9], coronary artery disease [10–11], stroke [12–13] and heart and kidney syndrome [14–15]. Various signal transduction pathways are involved in the EPO-promoted differentiation and proliferation of EPCs. AMP-activated protein kinase (AMPK) is essential for the differentiation of EPCs, and can activate eNOS activity and mediate the effects of vasculogenesis *in vivo* [16]. Similarly, a previous study demonstrated that AMPK transactivates eNOS through EPO [17]. Another study showed that EPO activates eNOS through Akt [18]. Evidence suggests that the EPO induction of NO is dependent on the expression of the βC receptor and the interaction of the βC receptor with vascular endothelial growth factor receptor type 2 (KDR) [19]. In addition, some studies have shown that EPO stimulates an increase in Ca^2+^_i_ through transient receptor potential cation channel subfamily C member 3 (TRPC3), but not TRPC6, and that the TRPC3 TRP domain and AMPK binding site are required for TRPC3 activation by EPO [20–21]. The JAK2-STAT5 axis is another essential pathway for EPO signaling. A prior study suggested that EPO-induced endothelial cell proliferation involves the STAT5 phosphorylation and nuclear translocation pathway [22]. Another study demonstrated that siRNA against redox-sensitive phosphatase SHP-2 restored EPO-mediated STAT5 induction confirming the contribution of the nicotinamide adenine dinucleotide phosphate-oxidase-2 (NADPH oxidase-2, Nox2) in EPCs [23]. The aforementioned signaling pathways activated by EPO are essential for the proliferation and differentiation of EPCs.

ROR (retinoic acid receptor-related orphan receptor) is an orphan nuclear receptor family comprising α, β, and γ subfamilies. Human vascular endothelial cells express only α1 and α4 subtypes [24–25]. Research has indicated that RORα is involved in the regulation of hypoxic signaling pathways [26], and studies conducted in different cells have shown that RORα nuclear receptors can activate AMPK [27–29]. Numerous studies have confirmed that RORα regulates circadian rhythm and metabolism [30–32]. In a previous study, we found that a natural compound bavachalcone (BavaC, CAS No.28448-85-3, derived from a traditional Chinese medical herb *Psoralea corylifolia* Linn) activates AMP-activated protein kinase activity and MnSOD expression [33] and induces RORα expression at the luciferase reporter, mRNA, and protein levels, partially inhibiting endothelial cell senescence [34]. However, the role of RORα in the proliferation and differentiation of EPCs is unknown. In this study, we investigated whether EPO promotes EPC differentiation by activating AMPK activity and whether RORα modulates EPO expression in cells stimulated with BavaC or a small molecule RORα activator.

## RESULTS

### BavaC promotes differentiation and cell recruitment of EPCs *in vitro*

**Figure 1:**
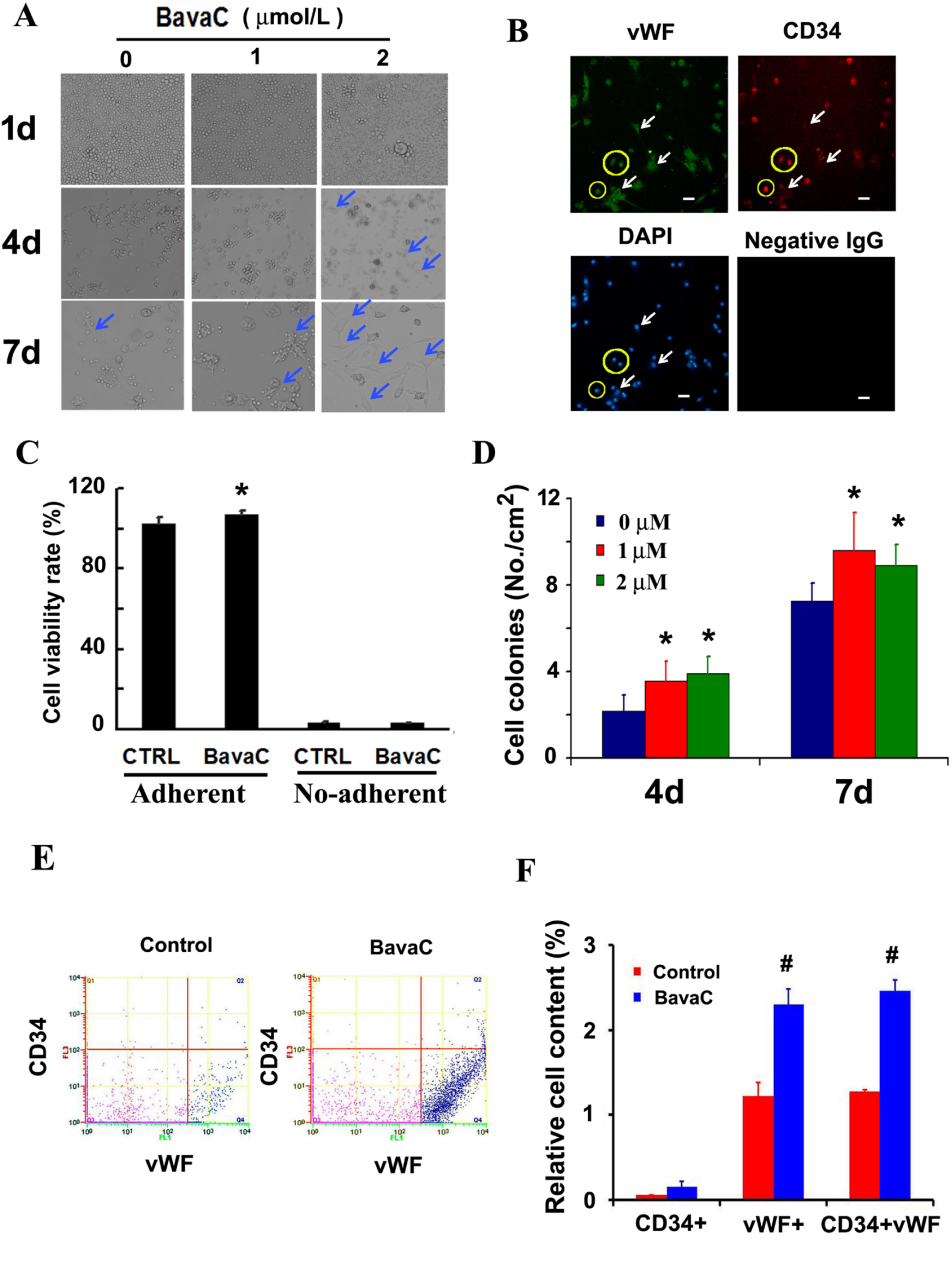
Effect of BavaC on differentiation of rat bone marrow stromal cells **(A)** The representative morphology of rat bone marrow stromal cell treated with 1 or 2 μM BavaC in the EBM-2 basal medium for 1, 4 and 7 days. Light blue arrows indicate fully adherent cells. **(B)** Rat bone marrow stromal cells treated with 2 μM BavaC for 7 days. Immunofluorescence staining involved anti-vWF (green) and anti-CD34 (red) antibodies. The surrounding cells in the yellow loop are differentiated endothelial progenitor cells, and white arrows indicate fully differentiated endothelial cells. Bar = 20 μm. **(C)** CCK-8 assay of rat bone marrow stromal cells treated or not treated with 2 μM BavaC in the EBM-2 basal medium for 7 days. Data are presented as mean ± SD, n = 5, *P < 0.05 vs. controls on the same date. **(D)** The number of colonies from Figure 1A in 35 mm diameter dishes. Data are presented as mean ± SD, n = 5, *P < 0.05 vs. negative controls on the same date. (**E** and **F**) The ratio of EPCs was determined using flow cytometry. Rat bone marrow stromal cells were treated with 1 μM BavaC in the EBM-2 basal medium for 7 days and, labeled with anti-vWF (green) or anti-CD34 (red) antibodies. Data are presented as mean ± SD, n = 3, *P < 0.05, total number of EPCs in the second and fourth zone vs. the control.

First, we confirmed whether EGM-2 medium promotes colony formation in resuspended non-adherent cells (bone marrow stromal cells) derived by culturing rat bone marrow in gelatin-coated dishes for 7 days (Figure 1A). The result showed that the EGM-2 medium promoted the differentiation of bone marrow cells into adherent cells. The suspended cells cultured in the medium containing 1 or 2 μM BavaC showed earlier adherence than the control group on the fourth and seventh days. They were stained through immunofluorescence by using anti-CD34 or anti-vWF antibodies, and the results confirmed that these cells differentiated into EPCs (Figure 1B). To determine whether the role of BavaC stimulates bone marrow cell growth or promote differentiation, we used CCK–8 cell assay to detect the effect of BavaC on bone marrow cells for 7 days. We found that BavaC only slightly promoted the number of adherent cells as compared to non-adherent cells, and the adherent cells increased to 106.77 ± 1.70% compared with 101.92 ± 3.21% for the control (n = 4, *P* < 0.05) (Figure 1C). In addition, BavaC promoted an increase in the cell colony number (colony-forming unit, CFU) on the fourth and seventh days; for example, on the seventh day, the cell colony number increased from 7.24 ± 0.83 CFU/cm^2^ in the control group to 9.60 ± 1.74 and 8.92 ± 0.93 CFU/cm^2^ in the BavaC-treated group (each n = 9, *P* < 0.05) (Figure 1D). To further determine whether BavaC promotes the differentiation of bone marrow stromal cells, antibodies against anti–CD34 and anti-vWF were used to label the cells cultured for 7 days in the medium containing 1 μM BavaC. The flow cytometry results showed that BavaC treatment led to an approximately 2-fold higher vWF/CD34^+^ EPC ratio (from 1.28% ± 0.01% up to 2.45% ± 0.13% of the total number of cells in the second and fourth zones) than that of the control group (each n = 3, *P* < 0.05; Figures 1E and 1F). Collectively, these data support that BavaC promotes the differentiation of rat bone marrow–derived cells into EPCs *in vitro*.

### BavaC improves vascular repair, and enhances hemodynamics and neovascularization *in vivo*

**Figure 2:**
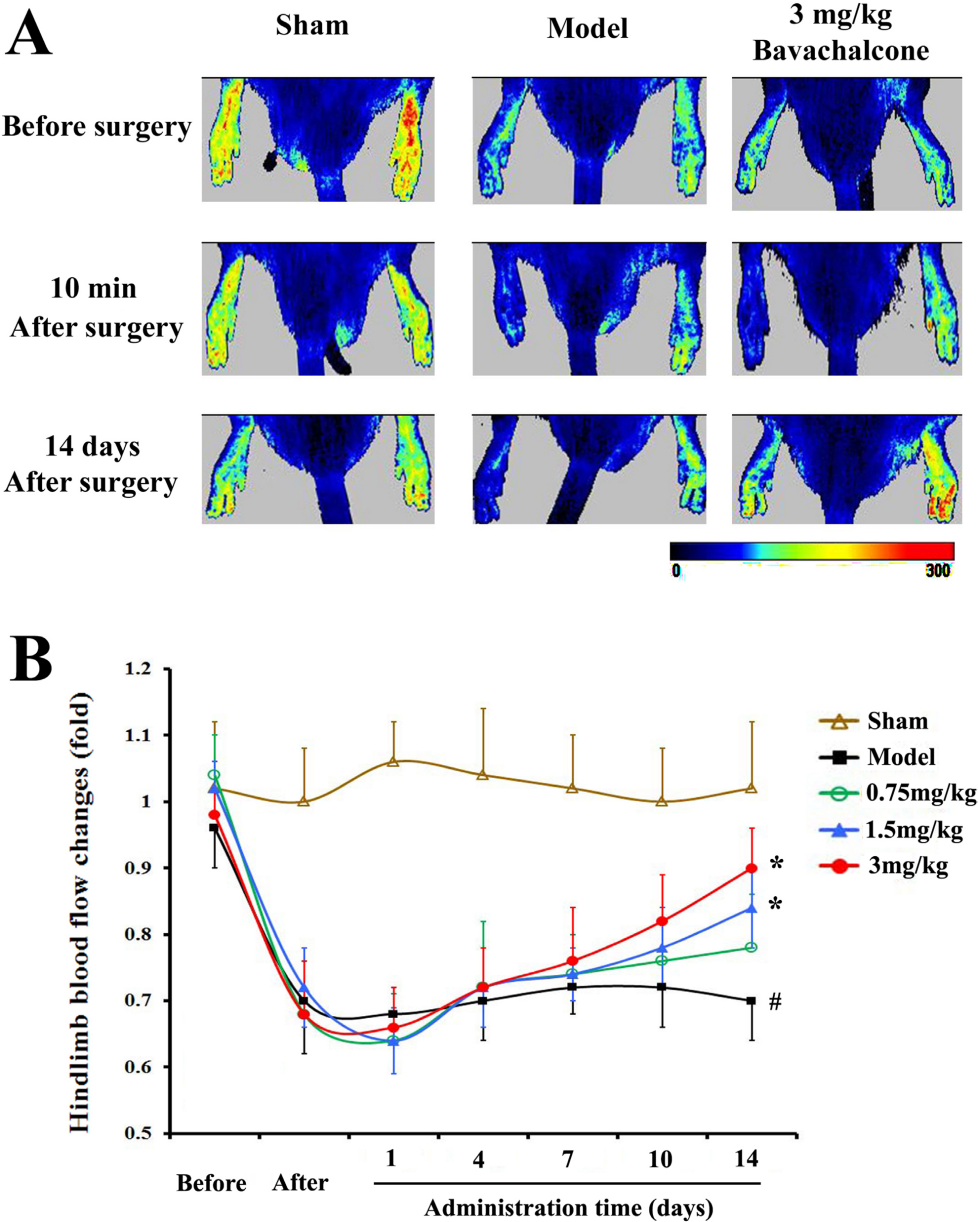
BavaC promotes blood flow restoration in the ischemic hindlimbs of the rats The Wistar rats in the sham operation group underwent sham operations on the left and right hindlimbs, whereas both ends of the femoral artery were ligated in the right hindlimb in the model and BavaC-treated groups, and the middle segment of vessels was cut off. Starting on the second day after the operation, the rats in the BavaC-treated group were given 3 mg/kg BavaC for 14 days by intragastric administration. The rats in the model and sham operation groups were given the adjuvant (CMC-Na) only. The effect of the operation on foot blood flow was confirmed using laser speckle flowmetry 10 min after surgery. Data are presented as mean ± SD, n = 5, #*P* < 0.05 vs. control group; *P < 0.05 vs. model group.

**Figure 3:**
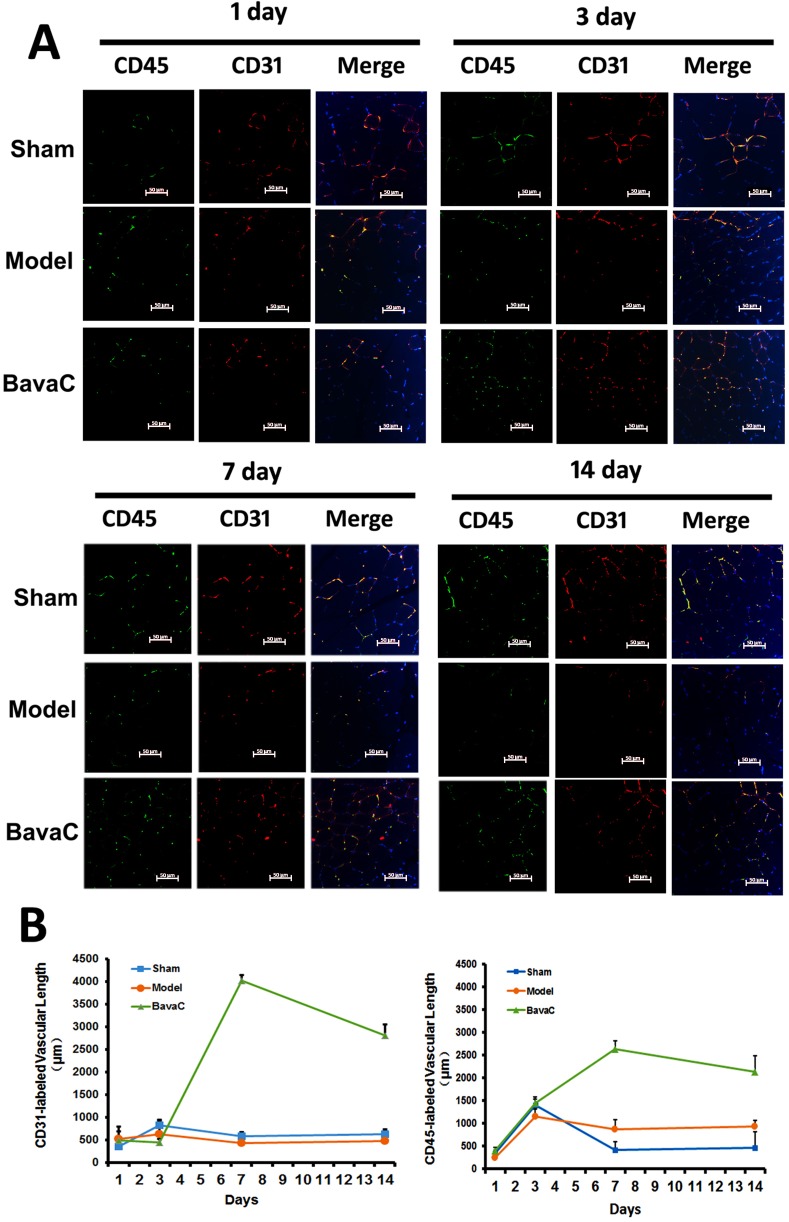
BavaC stimulates the neovascularization of capillaries derived from EPCs in ischemic muscles Immunofluorescence staining was performed on the right hindlimb muscle displayed in Figure 2. The muscle tissue sections were labeled with anti-CD31 (red) and anti-CD45 (green) antibodies. Cellular nuclei were stained with DAPI (blue). ImageJ Pro-Plus was used to calculate the lengths of the capillaries. Data are presented as mean ± SD, n=3, *P<0.05 vs. control group; # *P*<0.05 vs. sham-operation group. Bar = 50 μm.

**Figure 4:**
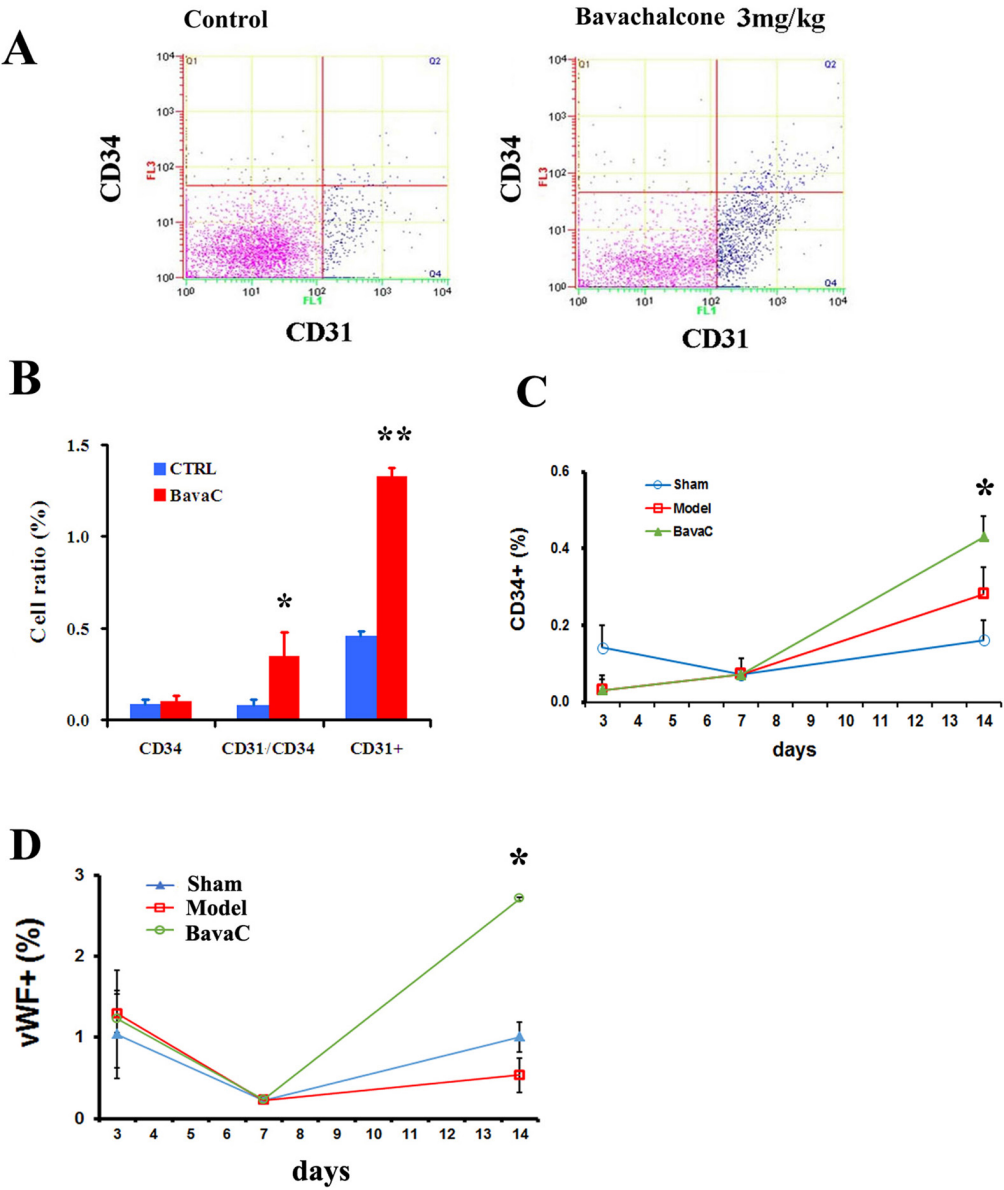
BavaC increases the circulating EPC ratio (**A** and **B**) Wistar rats in the BavaC-treated group were given 3 mg/kg BavaC for 7 days by intragastric administration. The rats in the control group were given the adjuvant (CMC-Na) only. Non-erythrocytes of rats were labeled with anti-CD31 (green) or anti-CD34 (red) antibodies, respectively, and analyzed by flow cytometry. Data are presented as mean ± SD, n = 3, *P < 0.05 vs. control group; ** *P* < 0.01 vs. control group. (**C** and **D**) Wistar rats were treated or not treated with 3 mg/kg BavaC for 1, 3, 7, and 14 days by intragastric administration. Non-erythrocytes were labeled with anti-vWF (green) or anti-CD34 (red) antibodies, respectively, and analyzed by flow cytometry. Data are presented as mean ± SD, n = 4, *P < 0.05 vs. model group.

To evaluate the effect of BavaC on EPC differentiation *in vivo*, we used the rat hindlimb ischemia model to test whether BavaC promotes the differentiation of EPCs. First, no significant differences were observed in the body weight, and the visceral coefficient of the heart, liver, and kidney of rats orally administered BavaC for 2 weeks (data not shown). Second, in the right hindlimb ischemia model of Wistar rats, we measured blood flow in the hind-paws by using a laser speckle flowmeter at 10 min postoperatively and 1, 4, 7, 10 and 14 days postoperatively. As shown in Figure 2A, laser speckle imaging revealed that compared with the left hindlimb model, the right hindlimb model was in the ischemic condition from immediately after surgery to the 14th day postoperatively. However, 14 days after the administration of 3 mg/kg BavaC, blood flow in the right hindlimb was effectively restored (Figure 2A). Simultaneously, the results showed that the intragastric administration of BavaC (0.75, 1.5 and 3 mg/kg) improved the recovery of blood flow in the right hindlimb in a time- and dose-dependent manner (Figure 2B). On the 14th day after surgery, the blood flow ratios of the right/left hindlimbs of each group were 1.00±0.08 (sham), 0.72±0.08 (model), 0.90±0.08 (3 mg/kg BavaC), 0.81±0.14 (1.5 mg/kg BavaC) and 0.79±0.08 (0.75 mg/kg BavaC; each n=5, Figure 2B). The blood flow ratios in the groups administered 3 and 1.5 mg/kg BavaC exhibited significant differences from that in the model group (*P*<0.05 for all; Figure 2B). Third, we performed immunofluorescence staining of CD45 (green) and CD31 (red) to analyze the neovascularization of the capillaries in skeletal muscle slices in the right hindlimb. Compared with the sham operation group, CD31-labeled vascular length did not change significantly on days 1, 3, 7 and 14 in the model group. The lengths of CD31-labeled blood vessels treated with 3 mg/kg BavaC has significantly increased by day 7 and day 14, reaching 4016.0 ± 131.5 μm and 2817.5 ± 232.6 μm per slice, respectively (each n = 3, *P* < 0.05; Figure 3). By contrast, 3rd days after the operation, the length of CD45-labeled vessels. By contrast, by the 3rd day after surgery, there was a small increase in the vessel lengths in all groups, and thereafter, the length of vessels in the sham operation and model groups returned to the basic level. The length of the vessel in the bavaC treatment group continued to increase, and by days 7th and 14th reached 2633.5 ± 188.7 μm and 2142.0 ± 346.4 μm per slice, respectively (each n = 3, *P* < 0.05; Figure 3). The CD45-labeled vascular length is shorter than the CD31-labeled vascular length, indicating that the neovascularization of the capillaries was derived partly from EPCs and partly from the proliferation of endothelial cells themselves. Fourth, to observe the changes in the number of circulating EPCs, we analyzed the percentage of CD31^+^, CD34^+^ and CD31^+^/CD34^+^ cells in the peripheral circulation of the rats through flow cytometry. The percentages of both CD31^+^ and CD31^+^/CD34^+^ cells increased significantly after 14 days of BavaC administration (3 mg/kg) compared with the control group, from 0.45 ± 0.02% (CD31+) and 0.07 ± 0.03% (CD31/CD34) to 1.32 ± 0.04% and 0.34 ± 0.13%, respectively (each n = 5, *P* < 0.05, Figure 4A and 4B). The dynamics of circulating EPCs were also observed at different times. The number of circulating CD34^+^ EPC cells in the model group increased gradually, reaching 0.28 ± 0.07% on the 14th day, compared with 0.16 ± 0.05% for the sham operation group. Furthermore, the stimulation of BavaC further promoted the increase in the number of circulating CD34^+^ EPC cells, reaching 0.43 ± 0.05% on the fourteenth day (n = 3, *P* < 0.05, vs. model group; Figure 4C). Similarly, BavaC also increased the number of circulating vWF+ progenitor cells, reaching 2.71 ± 0.02% on the 14nth day higher than the 1.01 ± 0.18% of the model group (n = 3, *P* < 0.05, vs. model group; Figure 4D). These results indicated that BavaC can enhance vascular repair, improve neovascularization in ischemic tissue, and promote blood flow restoration *in vivo*.

### BavaC facilitates the AMPK-mediated differentiation of EPCs

**Figure 5:**
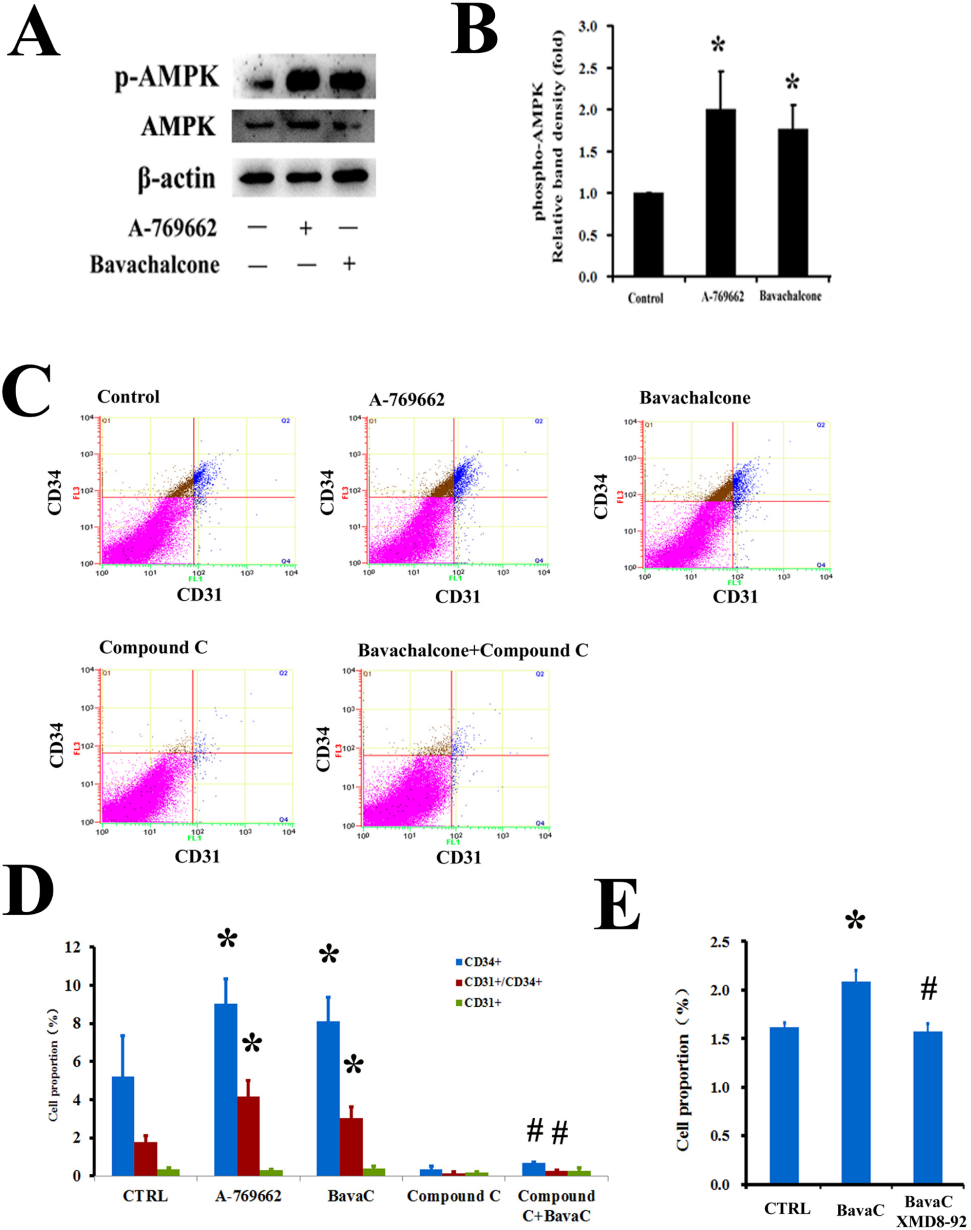
AMPK mediates the biological activity of BavaC in rat bone marrow stromal cells (**A** and **B**) The cells were incubated with the AMPK activator A-769662 or BavaC for 24 h. Protein phosphorylation and expression were assayed using Western blotting (n=3). Data are expressed as mean ± SD. *P < 0.05 vs. vehicle control; #*P* < 0.01 vs. treatment with BavaC alone. (**C** and **D**) The cells were incubated with the AMPK activator A-769662, AMPK antagonist Compound C, or BavaC or **(E)** with the ERK5 inhibitor XMD8-92 for 7 days; the cells were then labeled with anti-CD31 (green) or anti-CD34 (red) antibodies, and the ratio of CD31+/CD34+ cells was measured using flow cytometry. Data are presented as mean ± SD, n = 3, *P < 0.05, total number of EPCs in the second and fourth zones vs. the control.

In a previous study, we found that treating HUVECs with BavaC for 24 h could activate AMPK and manganese-dependent superoxide dismutase (MnSOD) expression [33]. A previous study reported that AMPK is involved in the differentiation of EPCs [16]. Therefore, we examined the role of AMPK in BavaC-stimulated rat bone marrow cells. After the cells were incubated with BavaC at a final concentration of 2 μM for 48 h, AMPK phosphorylation levels were 1.7-fold higher than those of the control (n = 3, *P* < 0.05, Figure 5A and 5B). Similar to BavaC, the incubation of the cells with the AMPK activator A-769662 at a final concentration of 1μM also doubled AMPK activity (n=3, *P*<0.05; Figure 5A and 5B). We also examined the role of BavaC-stimulated AMPK in promoting the differentiation of EPCs. The percentages of CD31^+^, CD34^+^ and CD31^+^/CD34^+^ cells were measured using flow cytometry on day 5 of the culturing of rat bone marrow-derived cells. With BavaC stimulation and A-769662 treatment, the CD31^+^/CD34^+^ cells increased from 1.78 ± 0.32% to 3.01 ± 0.60% and 4.17 ± 0.85%, respectively (each n = 3, *P* < 0.05; Figure 5C and 5D). However, Compound C, a potent selective and reversible AMP-kinase inhibitor, inhibited EPC differentiation stimulated by BavaC, and the percentage of CD31^+^/CD34^+^ cells was only 0.25 ± 0.03% (n = 3, *P* < 0.05; Figure 5C and 5D), suggesting that BavaC-induced AMPK activity is related to the differentiation of endothelial cells. Considering our previous finding that extracellular signal-regulated kinase 5 (ERK5) is the downstream signal molecule of AMPK [35], we evaluated the effect of ERK5 on EPC differentiation. The results showed that XMD8-92 at a final concentration of 5 μM, an inhibitor of ERK5, reversed BavaC-induced EPC differentiation, and the proportion of the cells decreased from 2.08 ± 0.11% to 1.57 ± 0.07%, which was similar to the 1.61 ± 0.04% of the control (n = 3, *P* < 0.05; Figure 5E). These results suggested that BavaC can modulate the differentiation of EPCs by activating the AMPK-ERK5 pathway.

### BavaC activates EPO expression *in vitro* and *in vivo* through nuclear orphan receptor ROR-α

**Figure 6:**
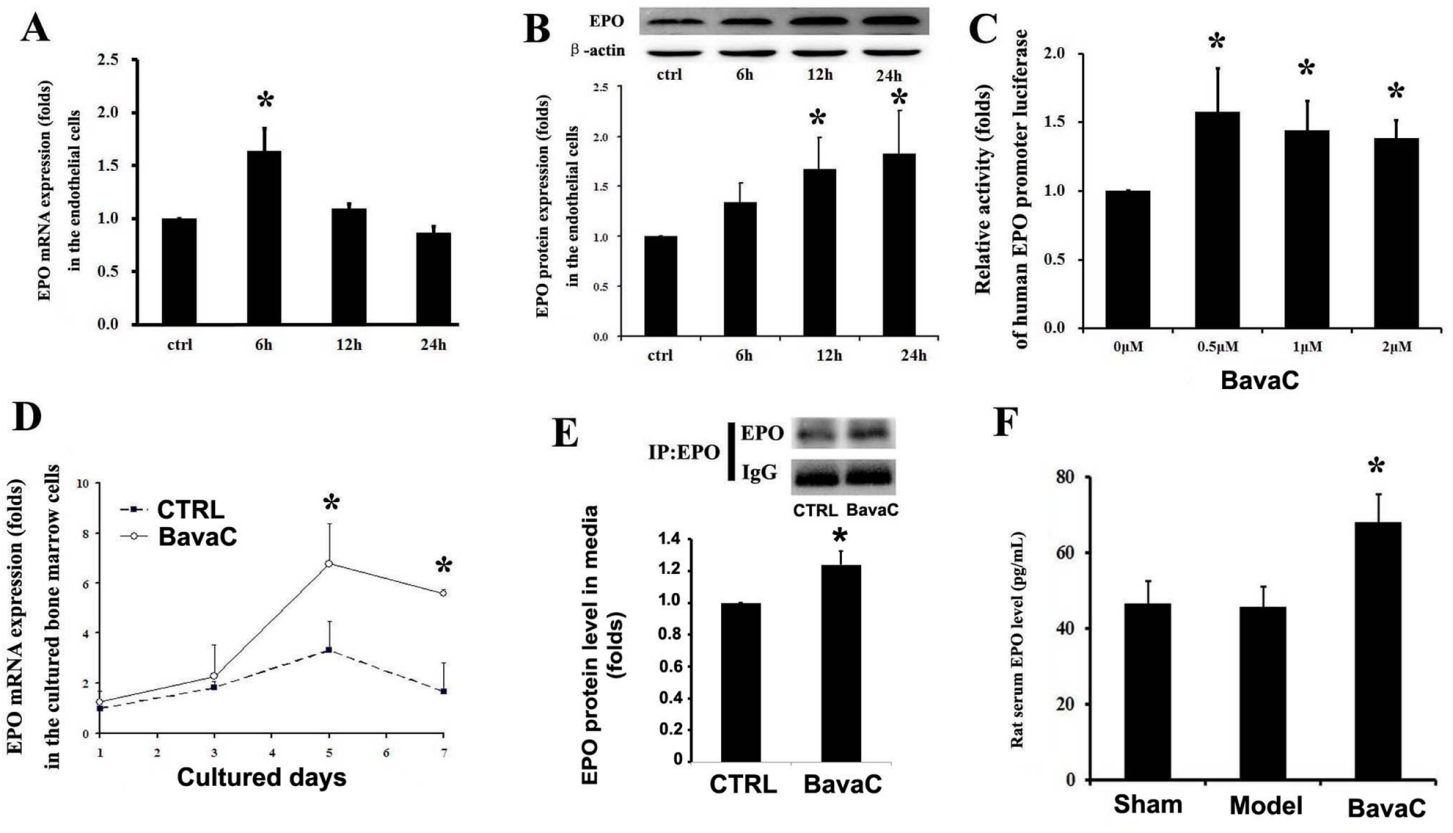
BavaC induces EPO expression and secretion (**A** and **B**) HUVECs were incubated with 2 μM BavaC for 0, 6, 12 or 24 h. EPO mRNA and protein expression levels were measured using real-time quantitative PCR or Western blotting (n = 3 each). Data are presented as mean ± SD, n=3, *P<0.05 vs. vehicle control. (**C** and **D**) Rat bone marrow stromal cells were incubated with BavaC at the indicated dose for 6 h or at 2 μM for the given days. EPO mRNA expression were measured using real-time quantitative PCR (n = 3 each). Data are presented as mean ± SD, n = 3, *P < 0.05 vs. vehicle control. **(E)** Rat bone marrow stromal cells were incubated with 2 μM BavaC for 1day. EPO protein in the cultured media was immunoprecipitated using EPO or control antibody and was determined using Western blotting. Data are presented as mean ± SD, n = 3, *P < 0.05 and **P < 0.01 vs. vehicle control. **(F)** The serum EPO levels of the rats from the experiment displayed in Figure 3 were determined using ELISA. Data are presented as mean ± SD, n = 5, *P < 0.05 vs. model rats.

The stimulation of the cells with BavaC for 24 h led to AMPK activation; thus, an unknown signal may participate in the regulation of EPC differentiation after the initiation of BavaC stimulation. We tested several factors for vascular endothelial cell growth and differentiation, including vascular endothelial growth factor and angiogenin-1. We found that stimulation with BavaC caused transient increases in EPO mRNA expression in HUVECs (n = 3, *P*< 0.05; Figure 6A). We also examined EPO protein expression in HUVECs through Western blotting and found a time-dependent, nearly 2-fold increase in protein expression at 24 h (n = 3, *P* < 0.05; Figure 6B). Thus, to confirm EPO action more clearly, we constructed a 700-bp length human EPO promoter luciferase reporter plasmid. After amplification and purification, the plasmid was introduced into EA.hy926 cells. After stimulation of the cells with 0.5, 1 and 2 μM BavaC, weak, approximately 1.5-fold reporter activity was found, which was not dose-dependent (n = 3, *P* < 0.05; Figure 6C). On the basis of these data, we investigated EPO mRNA expression in rat bone marrow stem cells incubated with BavaC. On day 5 of culturing, EPO mRNA expression reached a maximum level of nearly 7-fold and then decreased slightly (n = 3, *P* < 0.05; Figure 6D). We also collected the cultured media (the first and fifth days) of rat bone marrow stem cells, enriched the EPO proteins in the cultured media through immunoprecipitation, and determined their levels through Western blotting. As shown in Figure 6E, on the first day, the amount of secreted EPO protein in the cultured media after BavaC stimulation was significantly higher than that in the control group (n = 3, *P* < 0.05). From these observations, we further determined the EPO concentration in the serum of the preserved rat hindlimb ischemia model. The BavaC-treated (3 mg/kg) group, but not the model group, exhibited an increase in the serum EPO concentration, from 46.29 ± 6.12 pg/mL (control) and 45.20 ± 5.88 pg/mL (model) to 67.96 ± 7.47 pg/mL (n = 5, *P* < 0.05; Figure 6F).

**Figure 7:**
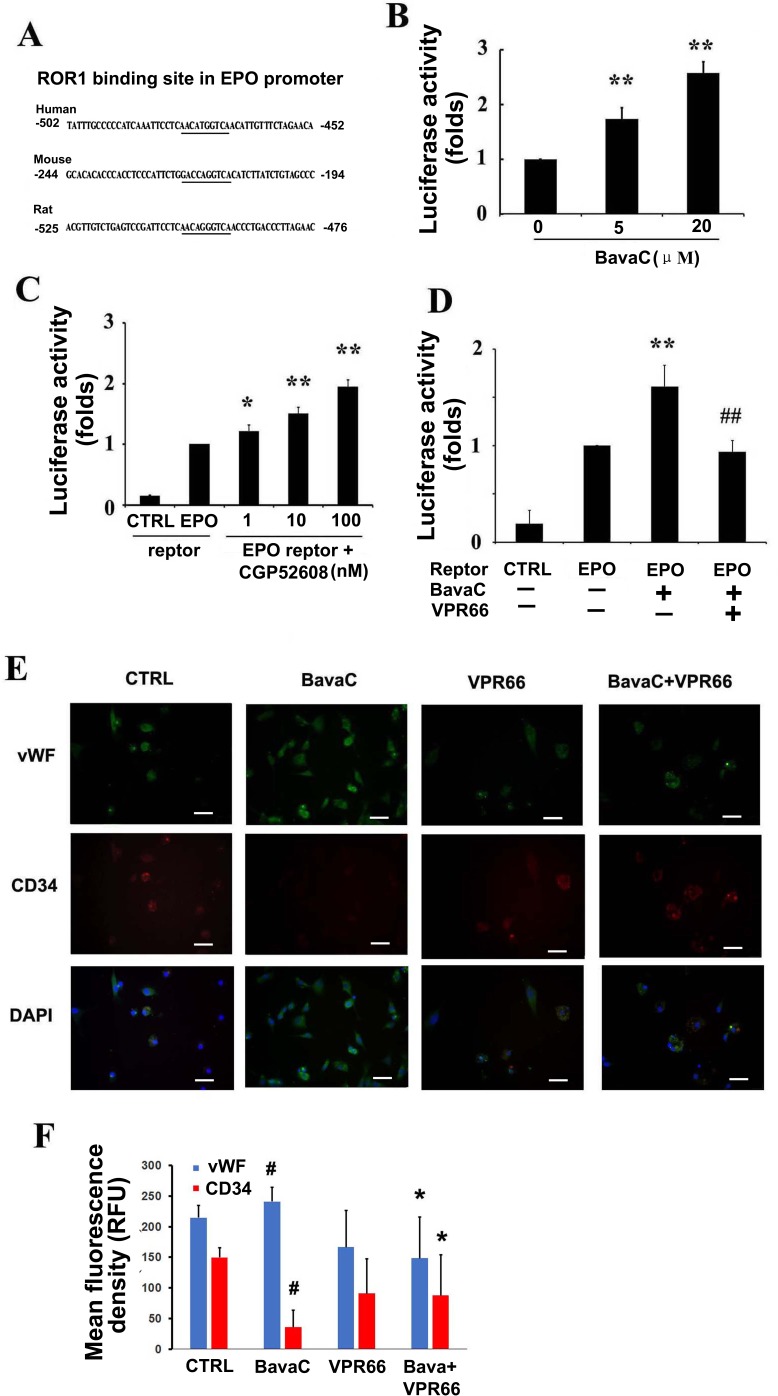

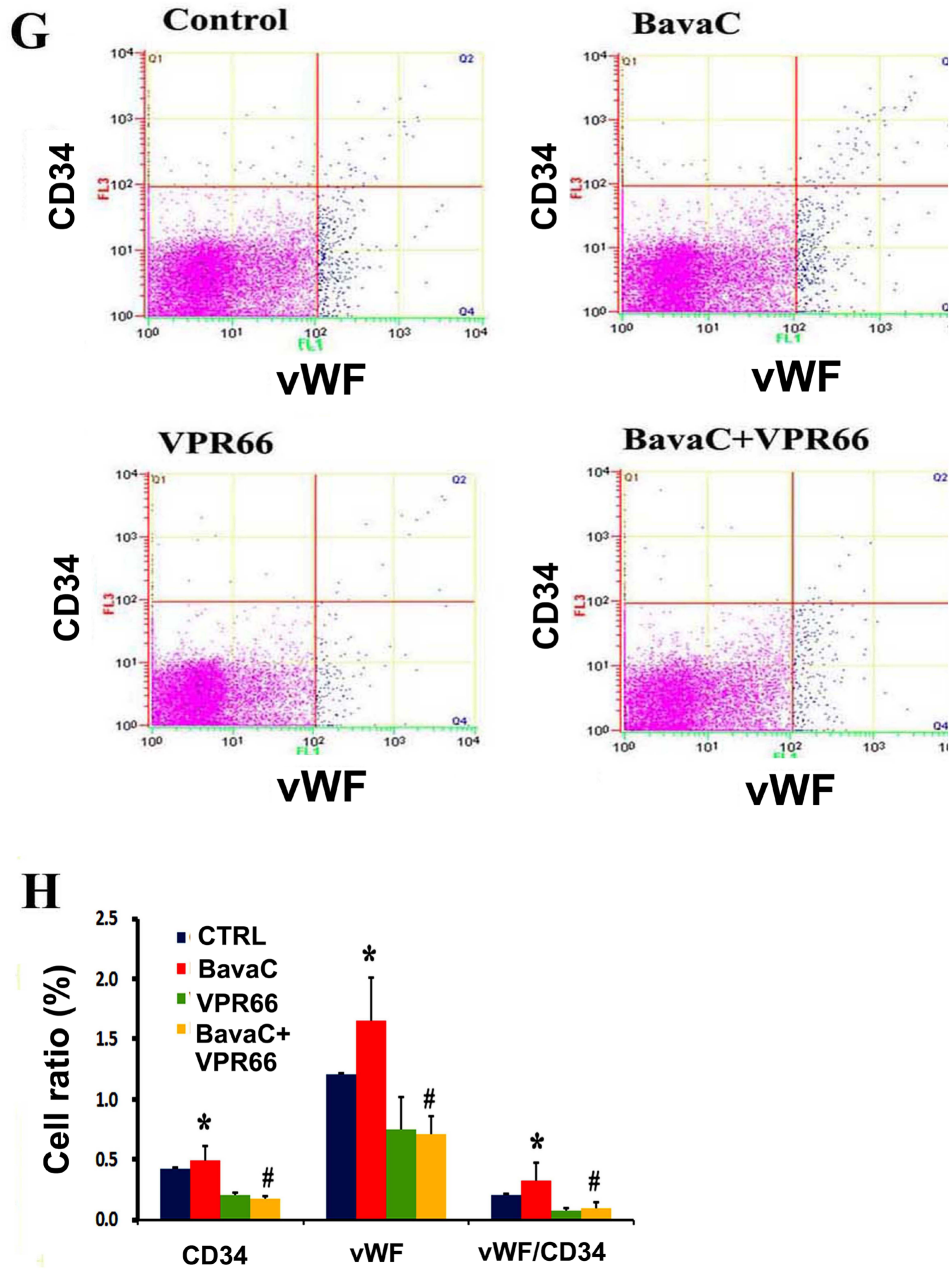
RORα1 mediates BavaC-stimulated EPO expression **(A)** RORα binding sites in the human, mouse, and rat EPO promoters. **(B)** Transient transfected EA.hy926 cells containing RORα (3×RORE) reporter luciferase plasmids were stimulated with the indicated doses of BavaC for 16 h, and RORα1 luciferase activity was measured (n = 3). Data are expressed as mean ± SD. **P < 0.01 vs. vehicle control. (**C** and **D**) Transient transfected EA.hy926 cells cells containing human EPO promoter reporter luciferase plasmids were stimulated with the indicated doses of the RORα activator CGP52608, or with 2 μM BavaC or BavaC plus the RORα antagonist VPR66 for 16 h, and EPO luciferase activity was measured (each n = 3). Data are expressed as the mean ± SD. *P < 0.05 vs. vehicle control, **P < 0.01 vs. vehicle control; ##*P* < 0.01 vs. BavaC alone. (**E** and **F**) Rat bone marrow stromal cells with or without 2 μM BavaC stimulation and/or with or without VPR66 treatment for 7 days. Immunofluorescence staining involved labeling with anti-vWF (green) and anti-CD34 (red) antibodies. Data are presented as mean ± SD, n = 3, *P < 0.05 vs. control group; #*P* < 0.05 vs. sham-operation group. Bar = 20 μm. **(G** and **H)** The cells were or were not incubated with 2 μM BavaC, and/or were incubated with VPR66 in the EBM-2 basal medium for 7 days. The cells were labeled with anti-vWF (green) or anti-CD34 (red) antibodies, and the ratios of CD34, vWF and vWF/CD34 cells were measured using flow cytometry. Data are presented as mean ± SD, n = 3, *P < 0.05, total number of EPCs in the second and fourth zones vs. the control.

We analyzed the human EPO promoter sequence and found that the orphan receptor RORα binding element was present; a similar binding element was also found in the rat and mouse EPO promoters (Figure 7A). In our previous study, we found that BavaC increased RORα expression [39]. To verify the previous results, we again determined the effect of BavaC on RORα1 luciferase reporter activity; the present findings were consistent with the previous findings (Figure 7B). CGP52608, a small molecule of the RORα activator, activated EPO promoter luciferase reporter gene activity in a dose-dependent manner, with a 2-fold increase at 100 nM (n = 3, *P* < 0.01; Figure 7C). Similarly, after BavaC treatment, EPO promoter reporter activity increased 1.5-fold (n = 3, *P* < 0.01; Figure 7D), By contrast, the pretreatment of the cells with VPR66, a RORα antagonist, at a final concentration of 10 μM for 1 h before BavaC treatment, substantially inhibited EPO promoter reporter activity compared with the BavaC-treated group (n = 3, *P* < 0.01; Figure 7D). Next, bone marrow cells were treated with BavaC in EGM-2 medium for 7 days, and immunofluorescence staining showed that BavaC stimulated vWF expression increased (from 214.74 ± 19.48 to 240.83 ± 23.24 RFU) and CD34 expression decreased (from 149.78 ± 15.35 to 35.88 ± 27.26 RFU) (n = 5, *P*< 0.05; Figure 7E and 7F), whereas the effect of BavaC on EPC differentiation was inhibited by VPR66 incubation with bone marrow cells for 7 days, and the relative fluorescence intensity of vWF and CD34 was reversed (vWF to 148.41 ± 67.51 RFU and CD34 to 87.71 ± 65.90 RFU, each n = 5, *P* < 0.05 vs. BavaC-treatment group; Figure 7E and 7F). Moreover, flow cytometry data showed the same trend. The VPR66 treatment reduced the CD34 cells from 0.49 ± 0.12% to 0.18 ± 0.02%; vWF cells from 1.65 ± 0.36% to 0.72 ± 0.14%, and vWF/CD34 cells from 0.32 ± 0.15% to 0.10 ± 0.05% (each n = 3, *P* < 0.05; Figure 7G and 7H). Overall, these results demonstrated that BavaC regulated EPO expression at the luciferase reporter, mRNA, and protein levels *in vitro* and increased the plasma EPO concentration *in vivo*, as well as caused EPC differentiation.

## DISCUSSION

The pharmacological stimulation of vascularization is an attractive treatment for ischemic disease. However, treatment with stem cells [36–37], EPO [8, 10] and granulocyte colony-stimulating factor [38–39], which increases the number of circulating EPCs and promotes the injury repair in the heart and other tissues, has many limitations, including high costs, short protein half-lives, immune rejection, and other side effects. Therefore, the development of low-cost and multi-effect pro-angiogenic agents is paramount.

A previous study showed that the consumption of red-wine rich in resveratrol (4-6 mg/L) reduced oxidative stress and increased capillary density by 46% in ischemic tissue [40]. In ApoE mice administered red wine, the number of EPCs increased by 60%, and their migration ability also significantly improved. Resveratrol exposure increased the activation of Akt/eNOS in endothelial cells [40]. Another study demonstrated the impairment of angiogenic activity in endothelial colony-forming cells from preterm infants (PT-ECFC), which also displayed a stress-induced senescence phenotype sustained by growth arrest and increased senescence-associated β-galactosidase activity. The nicotinamide adenine dinucleotide-dependent deacetylase activity of SIRTl was significantly reduced in PT-ECFC [41]. Transient SIRT1 overexpression or resveratrol treatment reversed the aging phenotype and rescued PT-ECFC angiogenesis defects *in vitro* in a SIRT1-dependent manner [41]. In addition, it has become clear that eNOS and MnSOD are essential for EPC differentiation, migration and antioxidant protection [42–45].

In this study, we found that BavaC could directly stimulate the differentiation of cultured rat thigh bone marrow cells into EPCs (Figure 1). Using the hindlimb ischemia models, we confirmed that BavaC at 3 mg/kg could promote the recovery of hindlimb blood flow after 14 days of intragastric administration (Figure 2). Fluorescence staining of the capillaries in the hindlimb muscles indicated that BavaC promoted angiogenesis in ischemic tissue, an effect derived from EPCs (Figure 3). One clear piece of evidence was that the CD45-labeled vascular length was shorter than the CD31-labeled vascular length, indicating that the neovascularization of the capillaries was derived partly from EPCs and partly from the proliferation of endothelial cells themselves. BavaC simultaneously increased the number of circulating EPCs, as determined through flow cytometry (Figure 4). This demonstrates that low-dose BavaC is effective for promoting the differentiation of EPCs.

Similar to resveratrol, we found that BavaC stimulated AMPK phosphorylation and activity only after the drug was added to the medium for 24 h [33]. In this study, we determined that BavaC and the AMPK activator A-769662 stimulated AMPK phosphorylation and the differentiation of EPCs in rat bone marrow cells (Figure 5), which was consistent with the findings in endothelial cells [33]. A previous study demonstrated that AMPK is essential for the differentiation of EPCs [16]. AICAR, an AMPK agonist, reinforces the positive effect of AMPK on EPC differentiation. The effects of AICAR on the angiogenesis of EPCs *in vitro* and *in vivo* were inhibited by treatment with Compound C [16]. A study reported that caffeine-rich coffee, rather than decaffeinated coffee, significantly increased EPC migration and increased serum caffeine levels in patients with coronary artery disease [46]. The treatment of a mouse model with caffeine for 7–10 days improved endothelial repair after denudation of the carotid artery. The enhancement of reendothelialization by caffeine was significantly reduced in AMPK knockout mice compared with wild-type mice [46]. We also found that XMD8-92, an inhibitor of ERK5, inhibited EPC differentiation, as revealed by flow cytometry (Figure 5E). Our previous results showed that resveratrol and pterostilbene stimulated AMPKα and ERK5 activity, and expression of MnSOD and KLF2, as well as reduce mitochondrial superoxide radical and endothelial cell senescence [35]. Prior studies have shown that ERK5 is an upstream signal molecule for KLF2 expression [35, 47], and a transcription factor that regulates eNOS and MnSOD expression [46, 48] and promotes EPC differentiation [49].

Because of the lag of AMPK activity after 24 h of BavaC stimulation in our results, we detected multiple secretory factors that promoted angiogenesis and found that EPO expression increased rapidly in endothelial cells and rat bone marrow-derived cells in mRNA, protein, and luciferase reporter levels (Figure 6). We found that EPO mRNA expression stimulated by BavaC was gradually enhanced peaking on the fifth day in bone marrow cells accompanying their differentiation process (Figure 6D), whereas BavaC stimulation for 16 or 24 h also induced a 1.5- to 2-fold increase in EPO luciferase activity and mRNA and protein expression in the HUVECs and EA.hy926 cells, respectively (Figure 6A-6C). This difference in expression may have been due to the need for EPO in bone marrow cell differentiation rather than in endothelial cells. On the basis of these findings, we examined the serum EPO concentration in the preserved rat hindlimb ischemia model. The results clearly demonstrated that BavaC increased the serum EPO concentration (Figure 6F). This evidence suggests that EPO is the initial active substance that enables BavaC to stimulate EPC differentiation. Several studies have reported that EPO activates EPC differentiation [5, 6] and AMPK activity [17, 20]. However, in previous studies [34] and the present study (Figure 7B), we demonstrated that BavaC activates RORα1. DNA sequence analysis revealed that the binding site of RORα is present in human and rat EPO promoters (Figure 7A). As shown in Figure 7C, the RORα activator CGP52608 could activate EPO luciferase reporter activity. However, the RORα antagonist VPR66 could inhibit BavaC-activated EPO promoter luciferase activity (Figure 7D) and EPC differentiation (Figure 7E-7H). This is the first time that EPO has been demonstrated to be regulated by RORα. Our results and those of other studies have suggested that RORα is likely to be an essential regulatory factor involved in many cellular and tissue differentiation and development processes, and the involvement of this factor is not confined to a few of the aforementioned cells.

**Figure 8:**
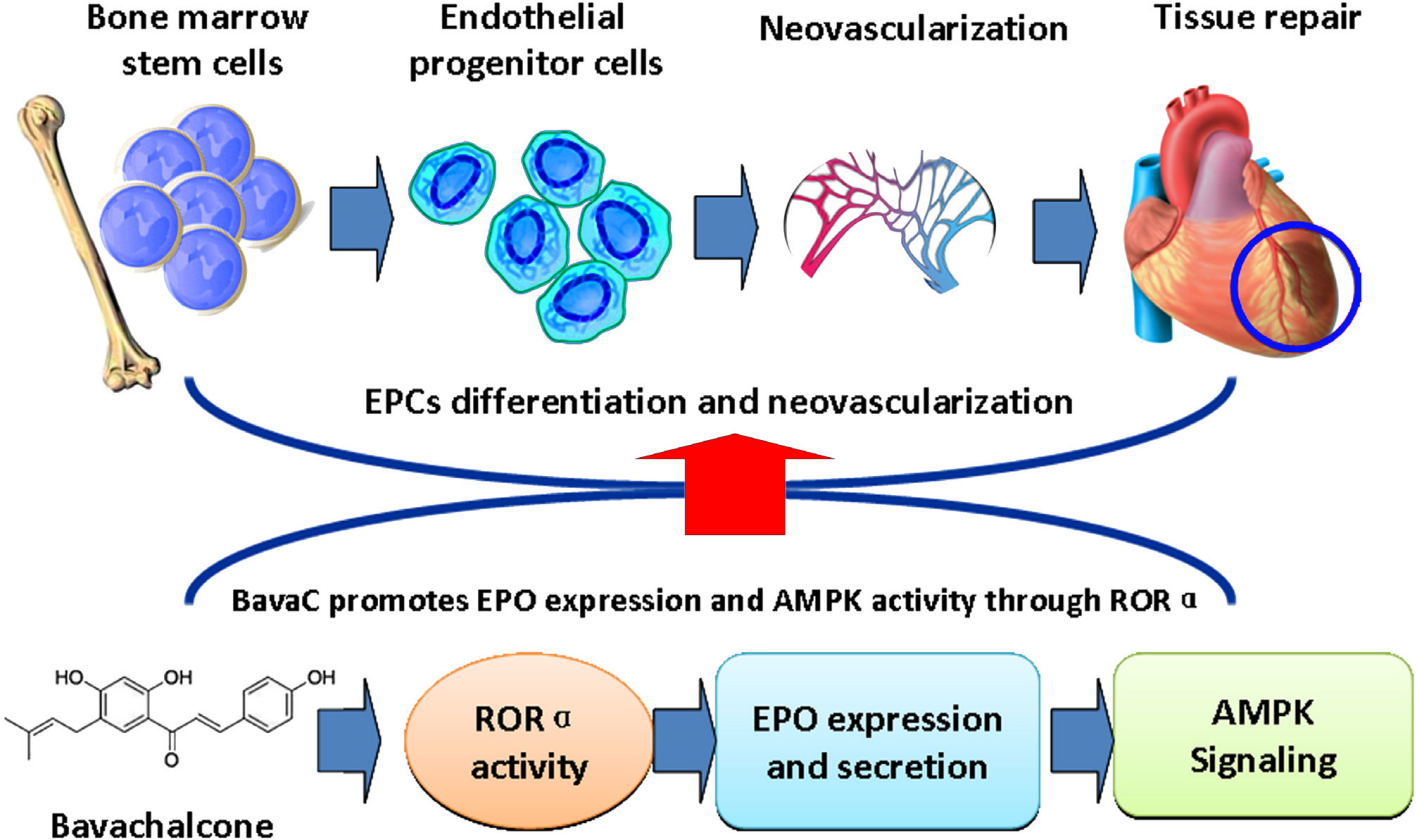
Schematic model demonstrating that BavaC promotes the differentiation of bone marrow cells into endothelial progenitor cells via the ROR-EPO-AMPK pathway

In conclusion, this study demonstrated that BavaC induced differentiation of rat bone marrow cells into EPCs. Administration of BavaC promoted the recovery of blood flow in ischemic hind limbs, and increased the number of circulating EPCs and the capillaries of ischemic hind limb muscle. We made the following discoveries: (1) BavaC treatment activated AMPK and ERK5 in rat bone marrow cells, which were blocked by their inhibitors Compand C and XMD8-92S, respectively; (2) BavaC treatment also elevated EPO mRNA and protein expressions *in vitro* and *in vivo*, as well as, the circulating EPO levels in rats; (3) BavaC and RORα activator CGP52608 enhanced the activity of RORα1 and EPO luciferase reporter gene, whereas the RORα antagonist VPR66 inhibited BavaC-induced EPO reporter activity and the differentiation of bone marrow cells into endothelial progenitor cells. These results are summarized in the schematic model in Figure 8. On the basis of the results of our study, we conclude that BavaC is a novel pro-angiogenic therapeutic agent that can be applied for effective systematic and specific tissue repair and regeneration in various ischemic diseases.

## MATERIALS AND METHODS

### Human endothelial cell cultures

Human umbilical vein endothelial cells (HUVECs) (CRL-1730) were purchased from ATCC (Manassas, USA), and EA.hy926 cells (GNHu39) were obtained from the Cell Resource Center of Shanghai Institute of Life Sciences, Chinese Academy of Sciences (Shanghai, China). HUVECs and EA.hy926 cells were maintained in a 37 °C incubator supplemented with 5% CO_2_ in Dulbecco’s modified Eagle’s medium (DMEM; Life Technologies, Carlsbad, USA) containing 10% fetal bovine serum. The medium was changed every 2 days and the cells were passaged with trypsin- ethylenediaminetetraacetic acid (EDTA). HUVECs were used in general pharmacological experiments *in vitro* and between 8–12 generations, and EA.hy926 cells were used in the luciferase reporter assay.

### Isolation and culture of rat bone marrow mesenchymal cells

Rat bone marrow mesenchymal cells were separated using density gradient centrifugation. After male Sprague Dawley rats (250±20g) were sacrificed, the femur and tibia were removed and immersed in prepared phosphate buffered saline (PBS). They were washed twice, both ends of the bones were cut off, and the bone marrow cavity was flushed into a centrifuge tube. The cells were centrifuged at 1000 rpm for 5 min at room temperature, the supernatant was removed, and the cells were transferred to Histopaque^®^-1083 density gradient medium (Sigma-Aldrich, St. Louis, USA) and centrifuged at 1500 rpm for 30 min at room temperature. The middle white blood cell layer in the centrifuged solution was transferred to a 15-mL centrifuge tube, mixed with 10 mL PBS, and centrifuged at 1000 rpm for 5 min at room temperature. The supernatant was discarded, and the cells were re-suspended by adding an appropriate volume of EBM-2 medium and were cultured. Non-adherent cells were collected and centrifuged at 1000 rpm for 5 min at room temperature. The supernatant was discarded, and the cells were cultured in a gelatin coated culture dish. Cell morphology was recorded every day, and the number of colonies was counted. Rat bone marrow cells were the only primary cells used.

### Hindlimb ischemia model and laser speckle contrast analysis

Male Wistar rats were purchased from Shanghai SLACK Laboratory Animal Co., Ltd. (Shanghai, China). These rats were handled in compliance with the Guide for the Care and Use of Laboratory Animals, and the animal experiments in this study were approved by the Animal Ethics Committee of Shanghai University of Traditional Chinese Medicine. For hindlimb ischemia, 8-week-old rats were anesthetized by intraperitoneal injection of 3% pentobarbital sodium (30 mg/kg), and their body temperature was maintained for 10 min on a thermal pad. Before surgery, the baseline blood flow was measured through laser speckle flowmetry with PeriCam PSI System (Perimed AB, Stockholm, Sweden). A small incision was made in the hindlimb near the groin to isolate the femoral artery. The rats in the sham operation group underwent sham operation on the left limbs, and ischemia was induced in the right limbs of the rats in the ischemia group. In the ischemia group, both ends of the femoral artery were ligated to the middle segment, and the wound was sutured and rinsed with penicillin (100 000 units). After the operation, the rats were placed on the thermal pad for 10 min to recover the body temperature, and blood flow level was measured again. Blood flow was then monitored at 1, 4, 7, 10 and 14 days. The rats in the BavaC-treated group were treated with 3 mg/kg BavaC (purity> 99%; HPLC, Shanghai U-Sea Biotech Co., Ltd., Shanghai, China) daily through intragastric administration, and the rats in the sham-operation and model groups received adjuvant 0.5% CMC-Na solution.

### Immunofluorescence staining

Experimental hind limb muscle tissues were fixed with 4% paraformaldehyde, paraffin-sectioned, dewaxed and rehydrated. The sections were blocked with 1% bovine serum albumin in PBS at room temperature for 30 min. To detect CD31 and CD45 expression, the sections were incubated with anti-CD31 goat (1:500, AF3628, R&D Systems) and CD45 rabbit (1:500, ab10559, Abcam) polyclonal primary antibodies followed by incubation with donkey anti-goat (Alexa Fluor 555) and donkey anti-rabbit (Alexa Fluor 488) secondary antibodies (1:1000, Invitrogen). Moreover, 4’,6-diamidino-2-phenylindole (DAPI) was used as histological background control. Immunofluorescence imaging was manually performed with the 40**×** objective lens (camera: DP70, ISO: 200, and Tv: 10 sec) of Olympus IX71 or IX83 fluorescence microscope (Olympus, Tokyo, Japan).

### Flow cytometry

To quantify the number of EPCs, rat peripheral blood cells and cultured rat bone marrow stromal cells were analyzed using flow cytometry. All procedures were performed according to the manufacturer’s instructions. After 30-min incubation with FITC-conjugated anti-vWF (ab8822) or FITC-conjugated anti-CD31(ab33858) and phycoerythrin-conjugated anti-CD34 (ab187284) antibodies (all from Abcam), immunofluorescence-labeled cells were washed with PBS, fixed with 2% paraformaldehyde, and analyzed using the Cell Lab Quanta SC Flow Cytometer with MPL (Beckman Coulter Inc., Brea, USA). The sorted cells were compared with the matched isotype controls to determine the percentage of stained cells.

### Western blotting

After BavaC treatment, the cells were centrifuged and lysed in Triton/NP-40 lysis buffer containing 0.5% Triton X-100, 0.5% Nonidet P-40, 10 m mol/L Tris (pH 7.5), 2.5 mmol/L KCL, 150 m mol/L NaCl, 20 m mol/L β-glycerolphosphate, 50 m mol/L NaF, and 1 m mol/L Na_3_VO_4_. They were sonicated using the JY92-2D ultrasonic homogenizer (NingBo Scientz Biotechnology Co., Ltd, Zhejiang, China) and centrifuged at 10 000 ×g for 10 min. The supernatant was utilized for protein concentration measurement by using a protein assay kit (Bio-Rad, Hercules, CA, USA), and 30 μg of protein was separated through sodium dodecyl sulfate–polyacrylamide gel electrophoresis and transferred to nitrocellulose membranes (Pall China, Shanghai, China). The membranes were blocked overnight with 5% nonfat dried milk in a buffer containing 140 m mol/L NaCl, 20 m mol/L Tris-HCl (pH 7.5) and 0.1% Tween 20 and were incubated with the following primary antibodies: RORα rabbit polyclonal antibody (ab60134, Abcam) or anti-GAPDH monoclonal mouse antibody (KangChen Bio-tech Inc., Shanghai, China). Finally, the membranes were incubated with a horseradish peroxidase (HRP)-conjugated secondary antibody, at 4°C with gentle shaking overnight. The membranes were washed to remove unbound antibodies. Subsequently, the membranes were incubated with ECL immobilon Western chemiluminescent HRP substrate (WBKLS0500, Millipore, USA) and imaged using the chemiluminescence imaging system (Tanon-5200 Multi, Tanon Science & Technology Co., Ltd., Shanghai, China). The Western blotting experiments were performed in triplicate.

### Quantitative realtime polymerase chain reaction

Total RNA was extracted using TRIzol (Life Technologies) according to the manufacturer’s instructions. Real-time polymerase chain reaction (PCR) amplification and detection were performed using the SYBR Green qPCR SuperMix-UDG with ROX (Life Technologies) in a fluorescence thermal cycler (StepOne Real-Time PCR system, Life Technologies) according to the manufacturer’s protocol. Gene expression was normalized by using GAPDH as a reference gene. The following primers were used in our study: human EPO forward: 5′-TAT-GCC-TGG-AAG-AGG-ATG-GA-3′, reverse: 5′-AGA-GCC-CGA-AGC-AGA-GTG- GT-3′; rat EPO forward: 5′-AGA-ATG-AAG-GTG-GAA-GAA-CAG-G-3′, reverse: 5′-CCG- AAG-CAG-TGA-AGT-GAG-G-3′; human GAPDH, forward: 5′-GAT-CCC-GCT-AAC-ATC- AAA-TG-3′, reverse: 5′-GAG-GGA-GTT-GTC-ATA-TTT-CTC-3′; rat GAPDH, forward: 5′-AGA-CAG-CCG- CAT-CTT-CTT-GT-3′, reverse: 5′-TAC-TCA-GCA-CCA-GCA-TCA-CC-3′. The primers were synthesized by Shanghai Generay Biotech Co., Ltd (Shanghai, China).

### Luciferase reporter gene activity assay

The thymidine kinase (−83 to +91) 3× RORE [TCG-ACT-CGT-ATA-ACT-AGG-TCA-AGC- GCT-G] sequence or the human EPO promoter sequence (−700 to 1) was generated by inserting the corresponding annealed oligonucleotides into the Luc pGL3-basic plasmid to yield the RORα1 activity reporter luciferase plasmid or the EPO promoter reporter luciferase plasmid, respectively. EA.hy926 cells at a density of 2 ×10^5^ cells/well were cultured in 24-well plates. When 70%-80% confluence was reached, the cells were used for transfection. In each well, 0.8 μg of the reporter vector of the pGL3 plasmid firefly luciferase gene, along with 0.016 μg of the reporter pRL-SV40 plasmid of the Renilla luciferase gene for normalization, was transfected with lipofectamine 2000 diluted in Opti-MEM. After a 6 h transfection, the medium was replaced with normal DMEM. To detect the induction effect of BavaC or the RORα activator CGP52608 on RORα1 or EPO expression after incubation with BavaC or the RORα activator CGP52608 for 16 h, the activity of the luciferase reporter gene was assayed using a dual-luciferase reporter 1000 assay system and detected using a Varioskan Flash microplate spectrophotometer (Thermo Scientific, USA).

### CCK-8 cell proliferation assay

The cell proliferation assay was performed using Cell Counting Kit-8 (Beyotime Biotechenology, Nantong, China) in accordance with the manufacturer’s protocol. Firstly, the rat bone marrow mesenchymal cells were isolated as described previously. Then, 100-μL isolated cell suspensions in different groups were seeded into 96-well plates at a concentration of 2 × 103 cells/well and cultured for 7 days. Each group had three replicate wells. Before observation, the medium was transferred to a new 96-well plate, and 90 μL normal EBM-2 medium was added. Each well was treated with CCK-8 solution and EBM-2 medium mixtures at the ratio of 1:9, and the mixtures were incubated for 1–4 h. Then, the optical density (OD) was determined at 405 nm. According to the manufacturer's protocol, a formula was used to calculate the cell viability as follows: cell viability rate (%) = (BavaC OD/Ctrl OD) × 100.

### Rat EPO enzyme-linked immunosorbent assay

Serum EPO concentrations in the control and BavaC treated rats were estimated by using a rat erythropoietin enzyme-linked immunosorbent assay (ELISA) kit according to the manufacturer’s protocol (BioLegend, San Diego, USA).

### Statistical analyses

All cell experiments were repeated at least three times. All rats were randomly divided into groups of at least five animals each. The results were expressed as means ± SD. The unpaired Student’s t test was used for the comparison of two independent groups, multiple comparisons were analyzed using one-way ANOVA followed by LSD. For all tests, *P* values < 0.05 were considered statistically significant.
